# Supplementary material for: Highly Charged Ru(II) Polypyridyl Complexes as Photosensitizer Agents in Photodynamic Therapy of Epithelial Ovarian Cancer Cells
Source: Int J Mol Sci. 2022 Nov 1;23(21):13302. doi: 10.3390/ijms232113302 (PMC9655950; doi:10.3390/ijms232113302)
Supplement: Supplementary file 1 [file ijms-23-13302-s001.zip › ijms-1966747-supplementary.pdf]

Supplementary Materials

Table of Contents

1. Acid-base properties of ruthenium compounds **Ru1**$^{2+}$ and **Ru2**$^{2+}$

2. Metal-binding properties of **Ru1**$^{2+}$ and **Ru2**$^{2+}$

3. Fluorescence properties of ruthenium compounds

4. ROS production and identification by [Cu**Ru1**]$^{4+}$ and [Cu$_2$**Ru2**]$^{6+}$

5. Redox properties of [Cu**Ru1**]$^{4+}$ and [Cu$_2$**Ru2**]$^{6+}$

6. Analysis of ruthenium compounds' distribution in mitochondria

7. Internalization of ruthenium compounds in cancer versus non-cancer cells

8. Analysis of ruthenium compounds' internalization in early endosomes

9. Cytotoxicity and photoactivity of Ru complexes in non-cancer cells

10. List of abbreviations

1. Acid-base properties of ruthenium compounds Ru1^{2+} and Ru2^{2+}

A preliminary requisite for the investigation of the binding properties of the receptor in its protonated forms concerns the knowledge of its basicity properties. The protonation equilibria of both **Ru1**$^{2+}$ and **Ru2**$^{2+}$ have been studied by means of potentiometric measurements in aqueous solution at 298.1 ± 0.1 K in 0.1 mol dm^{-3} NMe_4Cl at 298.1 ± 0.1 K. The protonation constants are reported in Table S1 and in Figures S1-S2 are shown the distribution diagrams of the protonated species present in solution.

[Ru(phen)$_2$**L′**]$^{2+}$ (**Ru1**$^{2+}$) and [Ru(phen)$_2$**L″**]$^{2+}$ (**Ru2**$^{2+}$) are capable of binding up to five ([H$_5$**Ru1**]$^{7+}$) and six ([H$_6$**Ru2**]$^{8+}$) protons in overall the range of pH investigated (between 2.5-10.5). In particular, it can be highlighted that in a wide range of pH, including at physiological pH value, the predominant species of ruthenium compounds present in solution are their di- and tetra-protonated forms, [H$_2$**Ru1**]$^{4+}$ and [H$_4$**Ru2**]$^{6+}$, respectively.

***Table S1**. Acid-base study of ruthenium compounds*

*Protonation constants of **Ru1**$^{2+}$ and **Ru2**$^{2+}$ determined by means of potentiometric measurements in NMe_4Cl 0.1 M, at 298.1 ± 0.1 K.*

**Reaction**	LogK **L**	
	**L = Ru1**	**L = Ru2**
$L^{2+} + H^+ = HL^{3+}$	9.50(4) [a]	11.23(8)
$HL^{3+} + H^+ = H_2L^{4+}$	7.59(5)	9.92(7)
$H_2L^{4+} + H^+ = H_3L^{5+}$	5.32(5)	8.37(5)
$H_3L^{5+} + H^+ = H_4L^{6+}$	3.91(5)	7.27(5)
$H_4L^{6+} + H^+ = H_5L^{7+}$	2.37(5)	
$H_4L^{6+} + 2H^+ = H_6L^{8+}$	-	7.13 (6)

[a] Values in parentheses are standard deviations in the last significant figure.

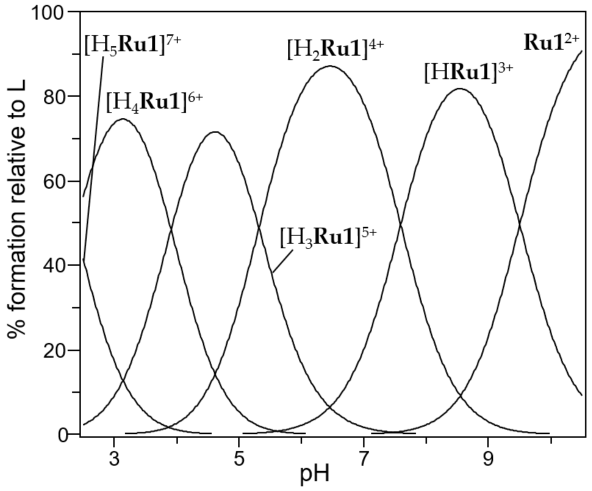

***Figure S1**. Distribution diagram of the protonated species of Ru1^{2+}*

*Distribution diagram of the protonated species of **Ru1**$^{2+}$ as a function of pH ([**Ru1**$^{2+}$] = 1 x 10^{-3} M, NMe_4Cl 0.1 M, 298.1 ± 0.1 K).*

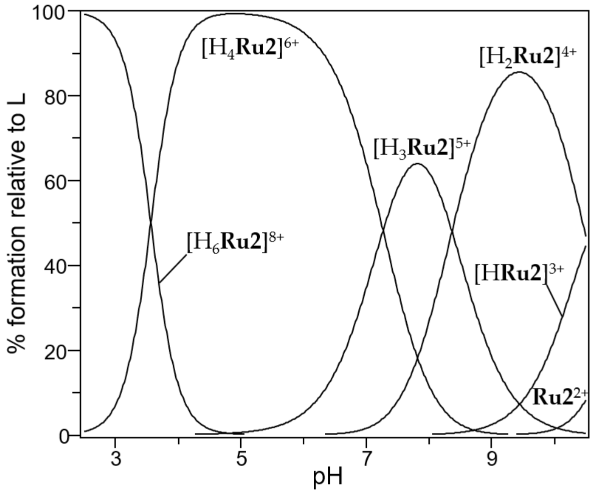

Figure S2. Distribution diagram of the protonated species of Ru2^{2+}

*Distribution diagram of the protonated species of **Ru2**$^{2+}$ as a function of pH ([**Ru2**$^{2+}$] = 1 x 10^{-3} M, NMe_4Cl 0.1 M, 298.1 ± 0.1 K).*

2. Metal-binding properties of Ru1^{2+} and Ru2^{2+}

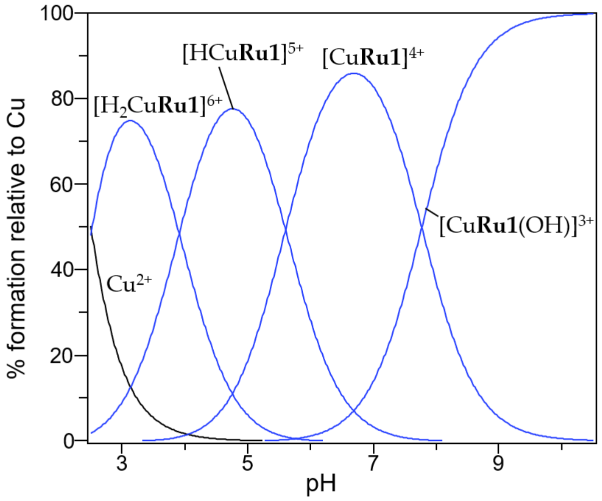

Figure S3. Distribution diagram of the Cu(II)-complexed species of Ru1^{2+}

*Distribution diagram of the species present in solution for the system **Ru1**$^{2+}$/Cu(II) in 1:1 molar ratio as a function of pH ([**Ru1**$^{2+}$] = [Cu(II)] = 1 x 10^{-3} M, NMe_4Cl 0.1 M, 298.1 ± 0.1 K).*

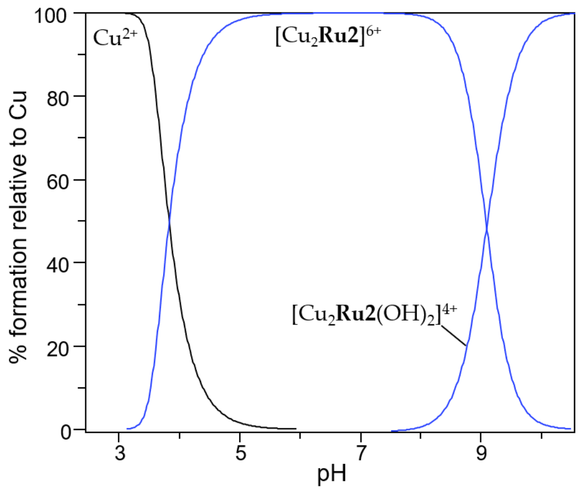

Figure S4. Distribution diagram of the Cu(II)-complexed species of Ru2^{2+}

*Distribution diagram of the species present in solution for the system **Ru2**$^{2+}$/Cu(II) in 1:2 molar ratio as a function of pH ([**Ru2**$^{2+}$] = 1 x 10^{-3} M, [Cu(II)] = 2 x 10^{-3} M, NMe$_4$Cl 0.1 M, 298.1 ± 0.1 K).*

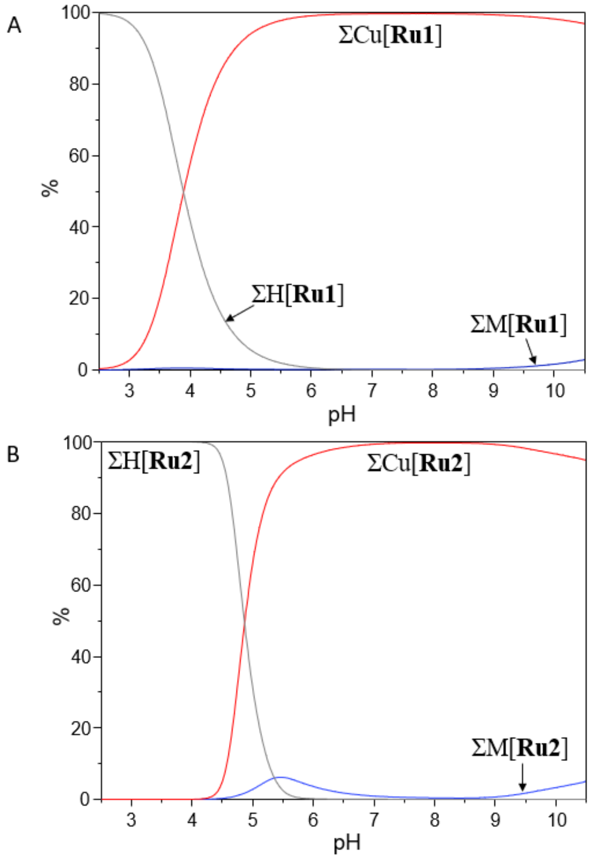

Figure S5. Selectivity diagrams with Cu(II) and other biologically relevant cations

*Overall percentages of the complexed species formed by **Ru1**$^{2+}$ (A) and **Ru2**$^{2+}$ (B) with Cu(II) (red lines), compared to the protonated forms of free ligands (grey lines) and to the metal complexed species formed with Ca(II), Zn(II) and Mg(II) (blue lines). [**Ru1**$^{2+}$] = [**Ru2**$^{+}$] = 1 μM, [Cu(II)] = 1 μM, [Zn(II)] = 15 μM, [Ca(II)] = 2.5 x 10^{-3} M, [Mg(II)] = 1.5 x 10^{-3} M.*

3. Fluorescence properties of ruthenium compounds

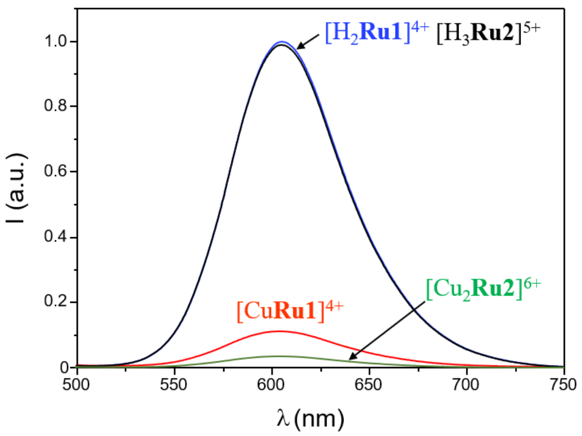

Figure S6. Fluorescence emission by Ru(II)- and mixed Ru(II)-Cu(II) complexes

Fluorescence spectra of aqueous solution of ***Ru1***$^{2+}$ *(blue line), [Cu**Ru1**]*$^{4+}$ *(red line),* ***Ru2***$^{2+}$ *(black line) and [Cu*$_2$***Ru2***]$^{6+}$ *(green line), collected at pH 7.4 (*λ_{exc} *= 411 nm, [Ru] = 3 μM). [H*$_2$***Ru1***]$^{4+}$*, [H*$_3$***Ru2***]$^{5+}$*, [Cu**Ru1**]*$^{4+}$ *and [Cu*$_2$***Ru2***]$^{6+}$ *are the main species present in solution at this pH value.*

4. ROS production and identification by [CuRu1]$^{4+}$ and [Cu$_2$Ru2]$^{6+}$

ROS production by [Cu**Ru1**]$^{4+}$ and [Cu$_2$**Ru2**]$^{6+}$ was performed by using Electron Paramagnetic Resonance (EPR) spin trapping technique. This technique involves the reaction of an initially generated short-living radical with an added organic compound, known as spin trap, to generate longer-living radical adducts with clear EPR fingerprints. Among the most common spin traps, 5,5-dimethyl-1-pyrroline-N-oxide (DMPO) efficiently captures the free radicals generated by an unstable species (H_2O_2) due to the catalysing action of a supposedly active molecule.

The spectra collected for a solution containing only H_2O_2 and DMPO, as reference sample, are reported, together with those obtained by adding [Cu**Ru1**]$^{4+}$ or [Cu$_2$**Ru2**]$^{6+}$ to this mixture, in Figure 1 of the manuscript. The acquired EPR spectra were also simulated by using the core function *pepper* of the open-source MATLAB toolbox EasySpin[1]. The simulations were obtained using an isotropic g factor = 2.0054 for both radicals and the following hyperfine constants: A = 42 MHz for both N and H nuclei in the hydroxide species and A = 32.5, 39.8, 3.4 MHz for H, N and H nuclei, respectively, according to literature[2]. The obtained results are shown in Figure S7.

It is important to notice that different concentrations of reagents (especially of the radical source, H_2O_2) can lead to different hydroxide/perhydroxyl radical ratios. All the EPR measurements reported in this paper were taken in the same experimental conditions and were therefore comparable.

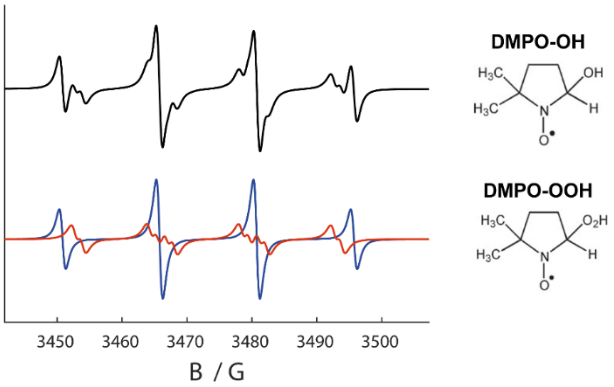

Figure S7**. **Simulation of the EPR spectra reported in Figure 1

The black trace is the best simulation obtained as the sum the EPR spectra of DMPO-OH (blue trace) and DMPO-OOH (red trace) radicals whose molecular structures are reported in the right part of the figure.

The lack of formation of singlet oxygen upon irradiation of the heteronuclear Ru(II)/Cu(II) complexes was confirmed through EPR measurements employing 2,2,6,6-tetramethylpiperidine (TEMP) as spin trap for single oxygen. In Figure S8 are reported the spectra obtained following irradiation of an aqueous solution of [Cu**Ru1**]$^{4+}$ at physiological pH in the presence of TEMP; similar results were also obtained for [Cu_2**Ru2**]$^{6+}$ (data not shown).

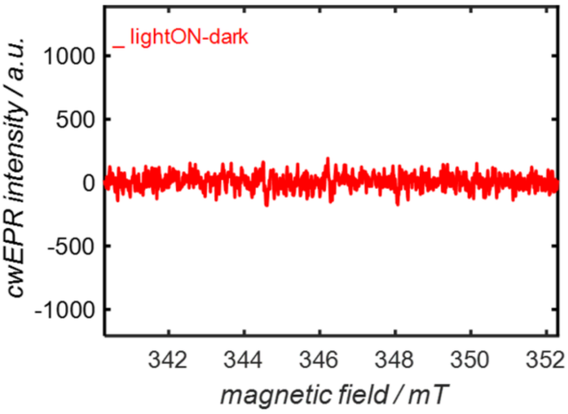

Figure S8. EPR analysis with TEMP as spin trap for singlet oxygen

*Difference between the spectra acquired under (lightON) and before (dark) light irradiation in a water solution (pH 7.4) containing [Cu**Ru1**]$^{4+}$ (5 mM) and TEMP (125 mM). For sample irradiation, a 405nm laser diode was used.*

5. Redox properties of [CuRu1]$^{4+}$ and [Cu_2Ru2]$^{6+}$

Redox potential, also known as oxidation-reduction potential (ORP), is a physicochemical parameter that can be used to measure the oxidative or reductive properties of a solution. ORPs of the [Cu**Ru1**]$^{4+}$ and [Cu_2**Ru2**]$^{6+}$ solutions were measured with an ORP electrode (HI3148B ORP, Hanna® Instruments, Padova, Italy) combined with a pH/ORP meter (HI5222 bench meter Hanna® Instruments, Padova, Italy). Before measurement, the electrode was calibrated with two redox standard solutions (HI7021 and HI7022, Hanna® Instruments, Padova, Italy). Glutathione (GSH) was obtained from Sigma-Aldrich.

Table S2*. *ORP analysis for heteronuclear complexes

*Oxidation-Reduction Potential (ORP) measured for 5 mL of 10 µM [Cu**Ru1**]$^{4+}$, 10 µM [Cu_2**Ru2**]$^{6+}$, and 5 mM GSH in 10 mM phosphate buffer pH 7.4.*

Sample	ORP value (mV)
[Cu_2**Ru2**]$^{6+}$	299.2 ± 4.9
[Cu**Ru1**]$^{4+}$	275.6 ± 1.3
GSH	130.0 ± 3.0

Table S2 shows the ORP values obtained for [Cu_2**Ru2**]$^{6+}$, [Cu**Ru1**]$^{4+}$ and GSH. By the analysis of these values, the ORP value of a 5 mM GSH solution turned out to be the lowest, thus confirming the higher reduction properties of this solution in comparison to both [Cu**Ru1**]$^{4+}$ and [Cu_2**Ru2**]$^{6+}$ solutions.

The redox properties of the two Cu(II)-containing complexes were also studied in cyclic voltammetry (CV) at a carbon screen-printed electrode. The redox system associated with changes in the oxidation state of copper ion is visible in the potential range from about +0.1 to -0.5 V, at a scanning rate of 50 mV/s. The potential-current parameters are reported in Table S3, including the cathodic peak potential ($E_{p,c}$), the anodic peak potential ($E_{p,a}$) and the ($E_{p,a}$+$E_{p,c}$)/2 values (the potential midway between the anodic and cathodic peaks, corresponding to the formal potential).

A lower potential value has to be reached on the cathodic wave to reduce the Cu(II) coordinated by [Cu**Ru1**]$^{4+}$ if compared to [Cu_2**Ru2**]$^{6+}$ (Table S3).

Nonetheless, given the highly reducing potential of GSH (Table S2), it cannot be excluded that the Cu(II) coordinated by **Ru1**$^{2+}$ and **Ru2**$^{2+}$ could be reduced in the cellular environment, where high concentrations of GSH are present.

***Table S3**. CV analysis for heteronuclear complexes*

*Potential of the anodic ($E_{p,a}$) and cathodic ($E_{p,c}$) peaks measured by cyclic voltammetry for 1 mM [Cu**Ru1**]$^{4+}$ and 1 mM [Cu_2**Ru2**]$^{6+}$ in 0.1 M KNO_3; scan rate 50 mV/s. KNO_3 was chosen as non-complexing supporting electrolyte. The solutions containing the two complexes were drop-casted (45 µL) onto the surface of disposable screen-printed electrodes (Ecobioservices and Researches (EBSR), Florence, Italy) composed of a carbon working electrode (Ø 3 mm), a carbon counter electrode, and an external Ag/AgCl reference electrode filled with saturated KCl. The voltammograms were recorded using a PGSTAT12 potentiostat/galvanostat running with GPES software (Metrohm Italia).*

Sample	$E_{p,a}$ (mV)	$E_{p,c}$ (mV)	($E_{p,a}$+$E_{p,c}$)/2 (mV)
[Cu**Ru1**]$^{4+}$	-40	-396	-218
[Cu_2**Ru2**]$^{6+}$	+14	-340	-163

6. *Analysis of ruthenium compounds' distribution in mitochondria*

Taking into account that the cationic nature of ruthenium compounds may affect their cellular localization and thus photoactivation-dependent mitochondrial depolarization, we evaluated possible localization of the photosensitizers in mitochondria. A2780 ovarian cancer cells were seeded on microscope slides and treated with 10 µM of each Ru complex for 24 hours. After incubation with 20 nM MitroTracker Green FM probe (#M7514, Thermo Fisher Scientific; Ex/Em: 490/516) in RPMI without phenol red for 30 minutes at 37°C, living cells were analyzed using a Leica SP8 laser scanning confocal microscope (Leica Microsystems GmbH) by 63X oil immersion objective.

Images of A2780 cells incubated with **Ru1**$^{2+}$, **Ru2**$^{2+}$, [Cu**Ru1**]$^{4+}$ or [Cu_2**Ru2**]$^{6+}$ and MitoTracker green are shown in Figure S9A. Scatterplots of red versus green pixel intensities of the images reported in Figure S9B show that a random distribution occurs between each ruthenium compound and mitochondria.

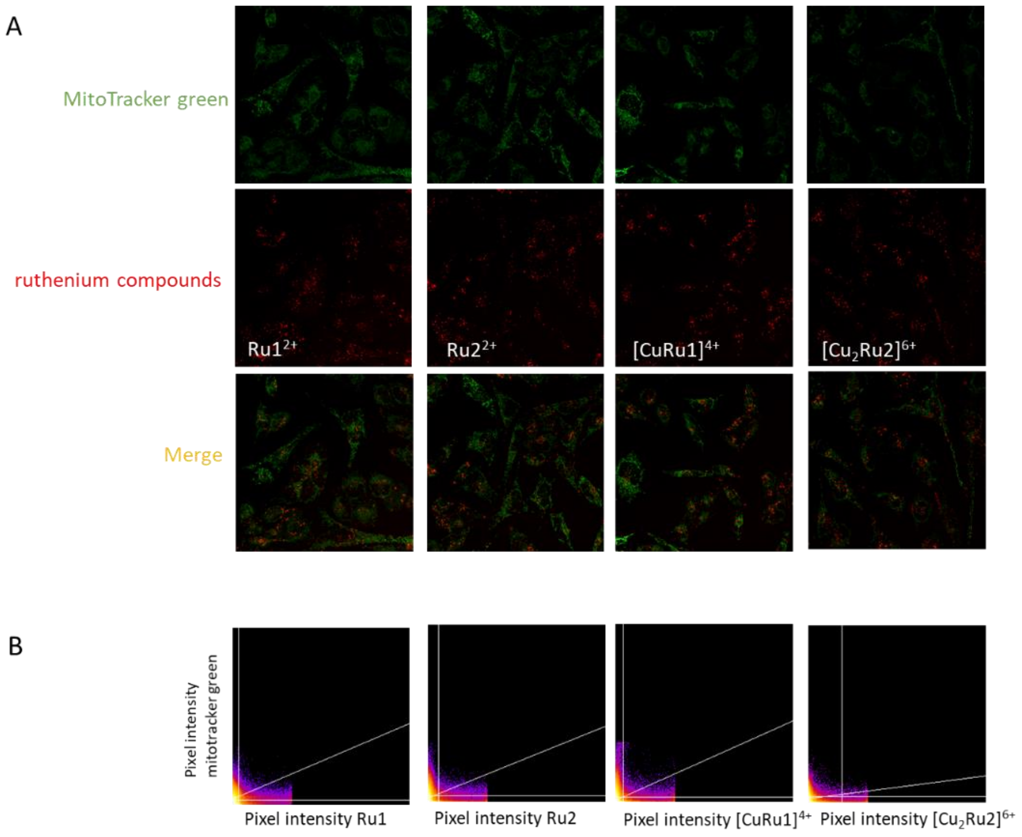

Figure S9*. *Localization of ruthenium compounds compared to mitochondria in A2780 cells

A) Confocal microscopy of A2780 cells following Mitotracker green (Green) staining after 24 h-incubation with ***Ru1***$^{2+}$*,* ***Ru2***$^{2+}$*, [Cu**Ru1**]*$^{4+}$ *or [Cu*$_2$***Ru2****]*$^{6+}$ *(Red). B) Scatterplots of red and green pixel intensities for each ruthenium compound.*

7. Internalization of ruthenium compounds in cancer versus non-cancer cells

In order to analyse ruthenium compound internalization in cancer versus non-cancer cells, we performed laser scanning confocal microscopy in non-cancer cell line, namely C2C12 myoblasts (undifferentiated skeletal muscle cells), as well as A2780 ovarian cancer cells, both incubated with 10 μM of each ruthenium compounds for 24 hours. Confocal microscopy images in Figure S10 show that in C2C12 myoblasts incubated with each photosensitizer the associated red fluorescence is not appreciable. However, in A2780 cells ruthenium compounds are located in segregated areas into the cytosol thus excluding passive diffusion as possible mechanism of ruthenium compound uptake.

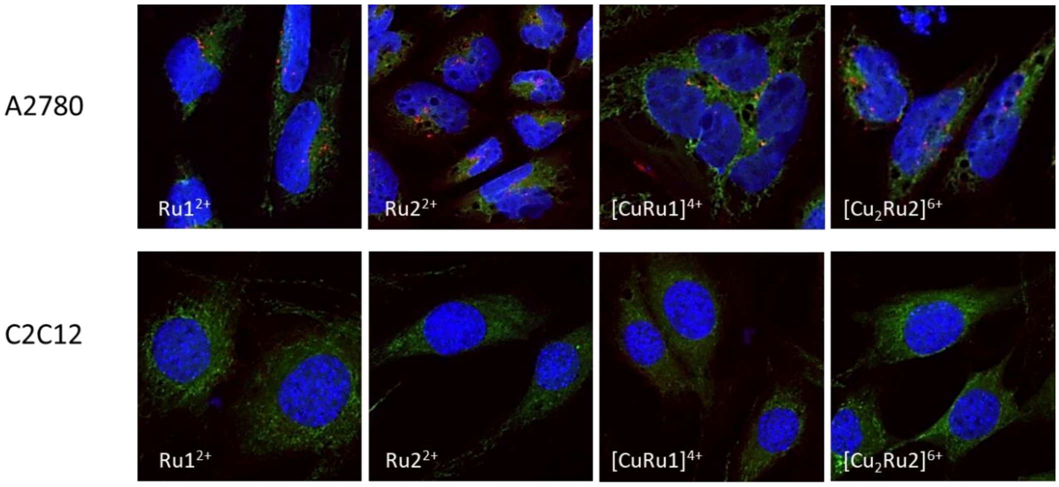

Figure S10. Internalization of Ru complexes in A2780 versus C2C12 cells.
A2780 ovarian cancer cells and C2C12 myoblasts were seeded into microscope slides, both incubated with each photosensitizer ***Ru1***$^{2+}$*,* ***Ru2***$^{2+}$*, [Cu**Ru1**]*$^{4+}$ *or [Cu$_2$**Ru2**]*$^{6+}$ *(Red) at a concentration of 10 μM for 24 hours and challenged with DAPI (Blue), after administration of Mitotraker CMX-Ros (Green). Confocal microscopy was performed to assess ruthenium compound internalization using Leica SP8 with a 63X objective.*

8. Analysis of ruthenium compounds' internalization in early endosomes

To evaluate the possible internalization pathways of ruthenium compounds in A2780 cancer cells, localization of **Ru1**$^{2+}$, **Ru2**$^{2+}$, [Cu**Ru1**]$^{4+}$ or [Cu$_2$**Ru2**]$^{6+}$ in early endosomes (EE) was analyzed. For this purpose, A2780 cells were seeded on microscope slides and treated with 10 μM of each Ru (II) complex for 30 minutes or 24 hours. After cell fixation using 2% paraformaldehyde, slides were incubated for 30 minutes with permeabilization and quenching solution (0.1% Triton X-100, 10 mM ethanolamine in PBS) and then with a blocking buffer (3% BSA in PBS) for 40 minutes. Subsequently, 1:100 Anti-Rab5 (E6N8S) Mouse Antibody (#46449, Cell Signaling Technology), maker of EE, was employed for 2 hours, then Fluorescein Anti-Mouse secondary antibody 1:200 (FI-2000) (Vector Laboratories, Inc., Burlingame, CA) was incubated for 1 hour at room temperature. Finally, DAPI solution (#MBD0015, Sigma-Aldrich) was administered to detect nuclei. Slides were mounted by using Fluoromount Aqueous Mounting Medium (Sigma-Aldrich, MA, USA) and images acquired using a Leica SP8 laser scanning confocal microscope (Leica Microsystems GmbH) by 63X oil immersion objective.
As reported in Figure S11, immunofluorescence analysis of Rab5 in A2780 cells after incubation with **Ru1**$^{2+}$, **Ru2**$^{2+}$, [Cu**Ru1**]$^{4+}$ or [Cu$_2$**Ru2**]$^{6+}$ for 30 minutes (A) and 24 hours (B) demonstrated negligible localization of ruthenium compounds with Rab5, ruling out the possible involvement of EE in the internalization pathway of Ru(II) photosensitizers.

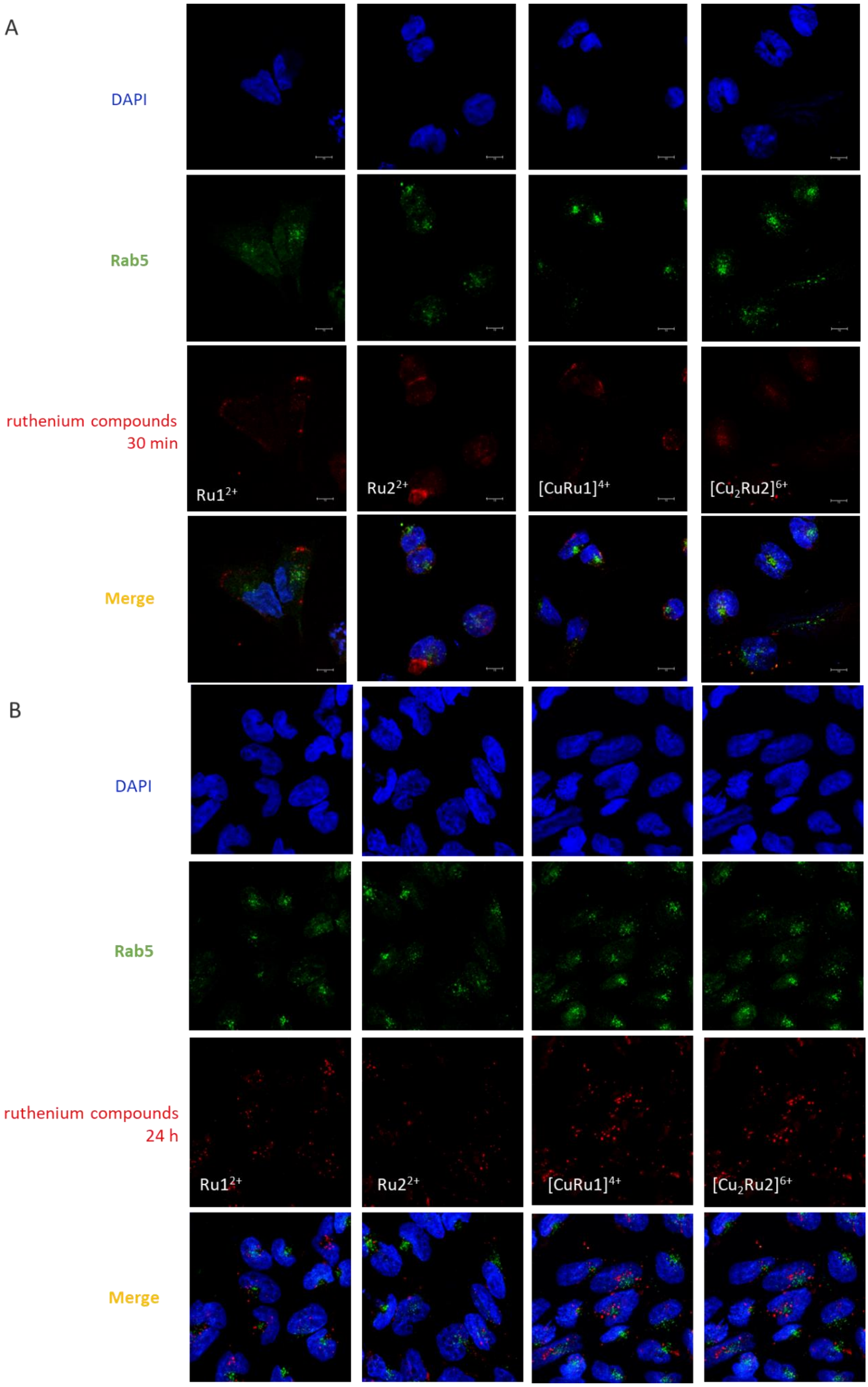

Figure S11. Localization of ruthenium compounds compared to early endosome in A2780 cells

*Immunofluorescence analysis of Rab5 in A2780 cells after incubation with 10 µM **Ru1**$^{2+}$, **Ru2**$^{2+}$, [Cu**Ru1**]$^{4+}$ or [Cu_2**Ru2**]$^{6+}$ for 30 min (A) or 24 h (B).*

9. *Cytotoxicity and photoactivity of Ru complexes in non-cancer cells*

To test cytotoxicity and photoactivity of different concentrations of **Ru1**$^{2+}$, **Ru2**$^{2+}$, [Cu**Ru1**]$^{4+}$ and [Cu$_2$**Ru2**]$^{6+}$ in non-cancer cells, MTT assays was performed in C2C12 myoblasts incubated for 24 hours with 0.1, 1 and 10 μM of each ruthenium compound then exposed or not to photoirradiation, 48 hours before being analysed. The photoirradiation experiments were conducted by using a low energy blue light-emitting diode (LED, λ_{max} = 434 nm, 30 W) and by employing the experimental set-up schematically represented in Fig. S12.

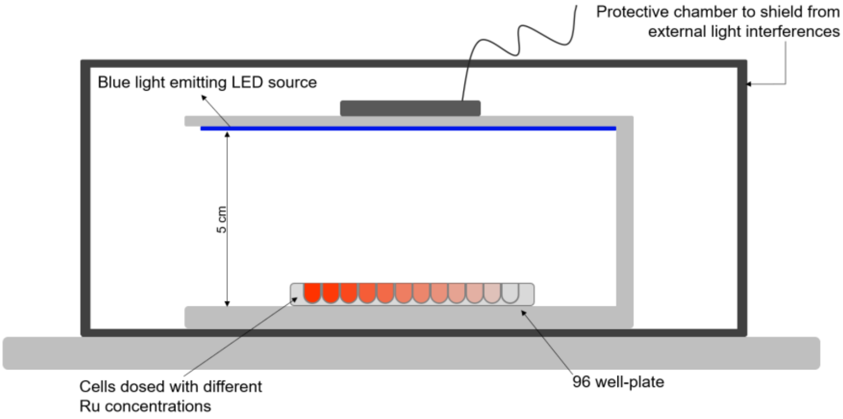

Figure S12. Experimental set-up employed for the photoirradiation experiments.

As shown in Figure S13, photosensitizers have a negligible effect on cytotoxicity of C2C12 myoblasts under dark conditions, whereas photoirradiation of **Ru1**$^{2+}$, **Ru2**$^{2+}$ or [Cu**Ru1**]$^{4+}$ shows a minor effect on cell survival of C2C12 myoblasts compared to A2780 cells that was not significant excepted for **Ru1**$^{2+}$, **Ru2**$^{2+}$, [Cu**Ru1**]$^{4+}$ at the higher (10 μM) concentration employed. The photoirradiation-induced cell death induced by [Cu$_2$**Ru2**]$^{6+}$ complex was not significant at all the concentrations used for MTT analysis.

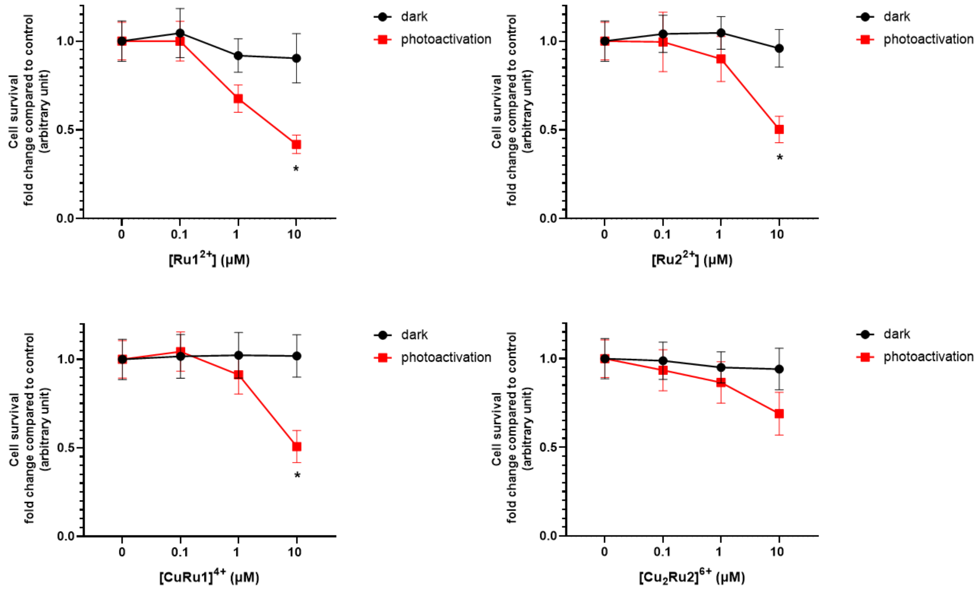

Figure S13. Cytotoxicity and photoactivity of ruthenium compounds in non-cancer C2C12 myoblasts.

*C2C12 myoblasts were seeded in 96 multiwell plates and MTT reduction assay was performed under dark or photoactivation condition after challenge with 0.1, 1 and 10 μM of each photosensitizer for 24 hours. MTT analyses were performed in pentaplicate and are representative of three independent experiments. Data reported are mean ± SD of fold change above control untreated. The effect of photoactivation of 10 μM of **Ru1**$^{2+}$, **Ru2**$^{2+}$, [Cu**Ru1**]$^{4+}$ and [Cu$_2$**Ru2**]$^{6+}$ complexes on cell survival of C2C12 cells was statistically significant by two-way ANOVA followed by Bonferroni post-hoc test: *P<0.05*

10. List of Abbreviations.

Photosensitizer agents	PSs
Photodynamic therapy	PDT
Reactive oxygen species	ROS
Ruthenium polypyridyl complexes	RPCs
Ground-state molecular oxygen	$^3O_2$
Singlet oxygen	$^1O_2$
Superoxide	$O_2^{\bullet -}$
Hydroxyl radical	$HO^{\bullet}$
Quantum yields	φ_Δ
Electron Paramagnetic Resonance	EPR
2,2,6,6-tetramethylpiperidine	TEMP
5,5-dimethyl-1-pyrroline-N-oxide	DMPO
Perhydroxyl	$HOO^{\bullet}$
Cyclic voltammetry	CV
Mitochondrial membrane potential	$\Delta\psi m$
Not significant	ns
CM-H_2DCFDA	DCF
Fetal bovine serum	FBS
Bovine Serum Albumin	BSA
Inductively Coupled Plasma Atomic Emission Spectrometer	ICP-AES
3-(4,5-Dimethylthiazol-2-yl)-2,5-Diphenyltetrazolium Bromide	MTT
Sodium dodecyl sulphate	SDS
Potential of the anodic peaks	Ep,a
Potential of cathodic peaks	Ep,c
Inductively Coupled Plasma Atomic Emission Spectrometer	ICP-AES
Phosphate buffered saline	PBS

References

[1] S. Stoll, A. Schweiger, EasySpin, a comprehensive software package for spectral simulation and analysis in EPR, J. Magn. Reson. (2006). https://doi.org/10.1016/j.jmr.2005.08.013.

[2] G.M. Rosen, A. Beselman, P. Tsai, S. Pou, C. Mailer, K. Ichikawa, B.H. Robinson, R. Nielsen, H.J. Halpern, A.D. MacKerell, Influence of Conformation on the EPR Spectrum of 5,5-Dimethyl-1-hydroperoxy-1-pyrrolidinyloxyl: A Spin Trapped Adduct of Superoxide, J. Org. Chem. (2004). https://doi.org/10.1021/jo0354894.
